# Oxymatrine ameliorates myocardial injury by inhibiting oxidative stress and apoptosis via the Nrf2/HO-1 and JAK/STAT pathways in type 2 diabetic rats

**DOI:** 10.1186/s12906-022-03818-4

**Published:** 2023-01-03

**Authors:** Yongpan Huang, Bin He, Chong Song, Xian Long, Jianbin He, Yansong Huang, Lijing Liu

**Affiliations:** 1 Medicine School, Changsha Social Work College, Changsha, 410004 Hunan China; 2 School of Nursing, Hunan University of Medicine, Huaihua, 418000 China; 3 Department of Respiratory and Critical Care Medicine, The First People’s Hospital of Huaihua, affiliated to University of South China, Huaihua, 418000 Hunan China

**Keywords:** Oxymatrine, Diabetes, Oxidative stress, Apoptosis, Myocardial injury, Inflammation

## Abstract

The necessity of increasing the efficiency of organ preservation has encouraged researchers to explore the mechanisms underlying diabetes-related myocardial injuries. This study intended to evaluate the protective effects of oxymatrine (OMT) in myocardial injury caused by type 2 diabetes mellitus. A model of diabetic rats was established to simulate type 2 diabetes mellitus using an intraperitoneal injection of a single dose of 65 mg/kg streptozotocin with a high-fat and high-cholesterol diet, and diabetic rats were subsequently treated with OMT (60, 120 mg/kg) by gavage for 8 weeks. Thereafter, diabetic rats demonstrated notable decreases in left ventricular systolic pressure (LVSP), ±dp/dt_max_, and in the activities of glutathione peroxidase, superoxide dismutase, and catalase. Moreover, we found notable increases in left ventricular end-diastolic pressure, fasting blood glucose, and malondialdehyde, as well as changes in cell apoptosis and decreased expression levels of Nrf2, HO-1, tyrosine protein kinase JAK (JAK), and signal transducer and transcription activator (STAT). Treatment with OMT alleviated all of the measured parameters. Collectively, these findings suggest that activation of the Nrf2/HO-1 and inhibition of the JAK/STAT signaling are involved in mediating the cardioprotective effects of OMT and also highlight the benefits of OMT in ameliorating myocardial injury in diabetic rats.

**Supplementary Information:**

The online version contains supplementary material available at 10.1186/s12906-022-03818-4.

## Introduction

Diabetic cardiomyopathy is a unique myocardial disease in patients with diabetes and is characterized by persistent hyperglycemia and cardiac dysfunction [1, 2]. During continuous hyperglycemia, the excessive production or insufficient removal of mitochondrial reactive oxygen species (ROS) in the body leads to the occurrence of oxidative stress, which is a key influencing factor for diabetic microangiopathy, and is also a pivotal cause of diabetic cardiomyopathy [3, 4]. Oxidative stress under hyperglycemic conditions contributes to reduced antioxidant capacity and also accelerates myocardial injury and initiates mitochondrial oxidative damage in diabetes mellitus, which deteriorates cell apoptosis and necrosis [5, 6]. Nuclear factor erythroid 2-related factor 2 (Nrf2), an important activator of antioxidant responsive element (ARE), regulates downstream target genes such as HO-1 [7, 8]. Nrf2, a key activator of ARE, which activates Nrf2 to dissociate from Kelch-like ECH-related protein 1 and enter the nucleus, forms a heterodimer with the macrophage activator protein in the nucleus, binds to the ARE sequence, and is subsequently regulated by ARE, which is a specific DNA-promoter binding sequence and an external regulatory region for the expression of phase II detoxification enzymes and cytoprotective protein genes. Previous studies have verified that the Nrf2/HO-1 pathway plays an important role in oxidative stress, which is also involved in myocardial injury [9, 10].

The tyrosine protein kinase JAK (JAK)/signal transducer and transcription activator (STAT) signaling pathway is an intracellular signaling pathway involved in the modulation of stress-response gene expression, by mediating signals from the cell surface to the nucleus, which is regarded as a pivotal player in the response to multiple cardiac injuries such as myocardial ischemia/reperfusion (I/R) injury. Inhibition of JAK/STAT signaling decreases the synthesis of TGF-β 1, the release of inflammatory mediators, and autophagy. According to a previous study, oxymatrine (OMT) suppresses JAK/STAT inflammatory signaling in gastric cancer through inhibition of the interleukin (IL)-21R-mediated JAK2/STAT3 pathway [11]. Moreover, OMT inhibits tumor growth and deactivates STAT5 signaling in a xenograft model [12].

OMT, the main component of *Sophora flavescens* Aiton, possesses extensive pharmacological effects, including antioxidant [13], antifibrotic [14], and anti-inflammatory properties [15]. Moreover, it has been widely accepted as a traditional remedy in myocardial ischemic injury, as well as in renal, liver, intestinal, and brain I/R injury in animal models [16]. OMT also exerts a significant effect on aortic endothelial dysfunction via oxidative stress in diabetic rats [17]. However, OMT has not yet been reported to offer myocardial protection in diabetes-related myocardial injury in rats, which is associated with the Nrf2-mediated antioxidant response. Therefore, using a model of myocardial injury in type 2 diabetic mellitus (T2DM) rats, the aim of this study was to investigate the potential effect of OMT on myocardial injury and elucidate the role of the Nrf2/HO-1 and JAK2/STAT3 signaling pathways in diabetic rats.

## Materials and methods

### Animals

Male SD rats weighing 160–200 g were purchased by the Experimental Animal Center of Changsha Social Work College. The rats were given free access to food and water. All experiments were carried out in accordance with the guidelines for the Care and Use of Laboratory Animals (NIH Publication No. 85–23, revised 1996 and Ethics Committees in Science: European Perspectives) and were approved by Changsha Social Work College Medicine Animal Care and Use Committee (XIANG 202109). All possible efforts were made to alleviate animal suffering.

#### Reagents and antibodies

Oxymatrine (purity > 98%) was purchased from Shanghai Aladdin Biochemical Technology Co., Ltd. Malondialdehyde (MDA) (cat.no. S0131M), glutathione peroxidase (GSH-Px)(cat.no. S0059S), superoxide dismutase (SOD)(cat.no. S0087), and catalase (CAT)(cat.no. S0082) were purchased from Beyotime (Shanghai, China). IL-6 and NF-κB (p65) were obtained from ELISA kits (R&D Systems Inc., Minneapolis, MN, USA; and MyBioSource, Southern California, San Diego, USA). Secondary antibodies, such as Alexa Fluor®488 labeled and Alexa Fluor®594 labeled goat anti-rabbit IgG (H + L), primary antibodies pSTAT3 (ab76315) were obtained from Abcam (Cambridge, UK). The primary antibodies STAT3 (bs-1658R and bsm-52235R) was purchased from Bioss. anti-Janus kinase 2(JAK2) (cat.no. 3230) and pJAK2 (cat.no. 3771) were obtained from Cell Signaling Technology. TUNEL staining kit was obtained from Sigma-Aldrich Co., Ltd.

### Diabetic rat model development

After 1 week of adaptive feeding, the animals were randomly assigned to one of the following groups: the control group (*n* = 10), T2DM group (n = 10), OMT (60 mg/kg) group (n = 10), or OMT (120 mg/kg) group (n = 10) [17]. The control rats were fed a regular diet, while the experimental group rats were fed a high-fat diet (consisting of 70% standard laboratory chow, 15% carbohydrate, 10% lard, and 5% yolk powder) as previously described [18, 19]. After 4 weeks of high-fat diet, the rats in the experimental group were injected with streptozotocin (STZ; 65 mg/kg, dissolved in 0.1 mol/L sodium citrate buffer; pH 4.4) intraperitoneally. The control group was intraperitoneally injected with a corresponding volume of citrate buffer (0.1 mol/L). Fasting blood glucose (FBG), oral glucose tolerance test (OGTT), and intraperitoneal glucose tolerance test (IPGTT) were conducted using venous blood samples to verify the establishment of a T2DM model. Rats in the T2DM groups were further randomly assigned into the OMT group (60 mg/kg) and the OMT group (120 mg/kg) (dissolved in saline) combined with a high-fat diet. Rats in the control and T2DM groups intragastrically received the same volume of saline once a day for 8 weeks.

### Measurement of left ventricular function index

The rats were anesthetized with an intraperitoneal injection of sodium pentobarbital (concentration, 3%; 60 mg/kg) [13, 20], and fixed on the operating table in the supine position, with an incision in the middle of the neck and endotracheal intubation. The right common carotid artery of the rat was separated and inserted into the left ventricle. The arterial cannula was connected to the pressure transducer, and also connected to the BL-420 N biological signal acquisition and analysis system (Chengdu Techman Instrument) in order to record the LVSP, the left ventricle, the ±dp/dt_max_ and LVEDP as previously described [21, 22]. Subsequently, venous blood was collected for biochemical analysis. All the rats were sacrificed by exsanguination following anesthesia, the tissues were collected for the following experiments.

### Histological examination

The hearts were collected and embedded in paraffin. The heart was sliced into 5-μm thick section, stained with HE and observed under a light microscope (Olympus Corporation). Histological examination was carried out in a blinded trial by an experienced pathologist. The scoring standard was recorded as follows: 0 indicated no damage, 1 indicated less than 25% damage, 2 indicated 25–50% damage, 3 indicated 50–75% damage, and 4 indicated more than 75% damage [18, 23].

### Evaluation of FBG, OGTT, and IPGTT

The FBG, OGTT, and IPGTT plasma concentrations were determined spectrophotometrically using a SpectraMax M5 instrument (Molecular Devices, LLC) and commercially available kits, following the manufacturer’s instructions.

### Determination of the levels of MDA, GSH-Px, SOD, and CAT

The supernatant from tissues was collected, and the activities of SOD, GSH-Px, MDA, and CAT were determined using the colorimetric kits [24].

#### Measurement of reactive oxygen species

The procedures of ROS measurement were according to manufacturer’s instructions. The tissue slices were incubated with DCFH-DA (cat# S0033, Beyotime, China) for 10 min at 37 °C. Following three washes with phosphate buffer solution for 1 min, the tissue slices were immediately photographed under an inverted fluorescence microscope. The mean fluorescence intensity was analyzed using the ImageJ software.

### Myocardial ultrastructural observation

Ultrastructural examination was performed as previously described [11]. Fresh rat myocardium was obtained and cut into 1-mm^3^ pieces, immersed in 2.5% glutaraldehyde at 4 °C and subsequently fixed. Sections were stained using uranyl acetate and lead bismuth citrate, and sections were detected using a transmission electron microscopy (Carl Zeiss AG).

### TUNEL staining

The levels of apoptosis were assessed using a commercial kit (cat. S7110, Sigma Aldrich). Briefly, tissues were excised and fixed in 4% paraformaldehyde in PBS at room temperature for 24 h. The fixed tissues were embedded in paraffin and stained. The numbers of apoptotic cells and total myocardial cells were counted in three randomly selected fields (magnification, × 200) under an immunofluorescence microscope. The apoptosis rate was defined as the mean percentage of apoptotic cells.

### Determination of IL-6 and NF-κB (p65)

Cardiac levels of IL-6 (cat: SRP4145, Sigma Aldrich) and NF-κB (p65) (cat:kt30141, Wuhan Merck Biotechnology Co., Ltd) were detected using ELISA kits in accordance with the respective manufacturers’ instructions.

### Reverse transcription-quantitative (RT-q)PCR

Total RNA was extracted using a RNeasy Mini kit and purified using 75% ethanol. The purified total RNA (200 ng/sample) was reverse transcribed into cDNA using a transcription kit. qPCR reactions were performed in triplicate using a SYBR® Green Master Mix and run on a LightCycler 480 system. The following thermocycling conditions were used: Pre-denaturation at 95 °C for 5 min; followed by 30 cycles of denaturation at 95 °C for 50 sec, annealing at 56.1 °C (Nrf2 and HO-1) and 59.4 °C (β-actin) for 50 sec, and extension at 72 °C for 50 sec; and a final total extension at 72 °C for 10 min. The following primers were used: Nrf2 forward, 5′-TTC CTC TGC TGC CAT TAG TCA GTC-3′ and reverse, 5′-GCT CTT CCA TTT CCG AGT CAC TG-3′, the length of the amplified product is 441 bp; HO-1 forward, 5′-ATC GTG CTC GCA TGA ACA CT-3′.

and reverse, 5′-CCA ACA CTG CAT TTA CAT GGC-3′, the length of the amplified product is 441 bp; and GADPH forward, 5′-TCC CAT CAC CAT CTT CCA-3′ and reverse, 5′-CAT CAC GCC ACA GTT TCC-3′, the length of the amplified product is 632 bp. The primers were synthesized by Guangzhou RiboBio. The relative gene expression was quantified using the 2^-∆∆Cq^ method [25].

#### Western blot

Protein was extracted from the myocardial tissue in RIPA buffer together with phosphatase and protease inhibitors (PMSF). The protein concentration was evaluated by BCA method. Tissue lysates were added with 4x Laemmlisample buffer, boiled and separated by SDS-PAGE. Proteins were transferred to a PVDF membrane (Bio-Rad, Hercules, CA). After nonspecific blocking with skim milk or BSA for 1 h, the membranes were probed with primary antibodies, including pSTAT3 (Abcam, 1:5000), STAT3 (Bioss, 1:1000), JAK2 (Cell Signaling Technology, 1:1000) and pJAK2 (Cell Signaling Technology, 1:1000) in 5 ml blocking buffer overnight at 4 °C. Next, the membranes were washed 4 times with Tris-buffered saline supplemented with 0.1% Tween 20 (TBST) and incubated with an appropriate HRP-conjugated secondary antibody. Membranes were washed 4 times with TBST, incubated with an ECL solution (Millipore) and d imaging was conducted using an BioRad imaging system.

### Statistical analysis

Statistical analysis was performed using GraphPad Prism 6.0 (GraphPad Software, Inc.), and data were expressed as the mean ± standard deviation. Statistical differences were assessed using Student’s *t* test between two groups, or using one-way ANOVA between more than two groups, followed by Tukey’s post-hoc tests. The level of statistically significant difference was set at *P* < 0.05.

## Results

### Diabetic rats exhibit exacerbated dysglycemia and cardiac dysfunction

**Fig. 1:**
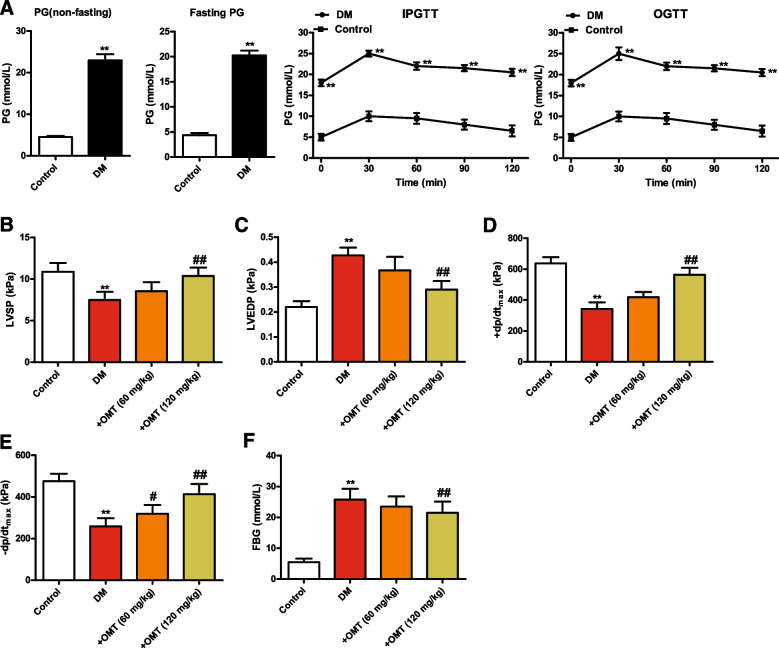
Confirmation of a diabetic rat model establishment. OMT attenuates ventricular hemodynamic parameters and FBG level in diabetic rats. **A** Non-fasting plasma glucose, fasting plasma glucose, IPGTT, and OGTT following 7 days of streptozotocin injection. **B** LVSP; **C** LVEDP; **D** + dp/dt_max_; **E** -dp/dt_max_; **F** FBG; Data are expressed as the mean ± standard deviation (*n* = 8). ^*^P < 0.05 compared with the control group. ^#^*P* < 0.05 compared with the diabetic group

Compared with the control group, diabetic rats showed significantly increased levels of non-fasting and fasting serum glucagon, accompanied by markedly impaired non-fasting PG, fasting PG, IPGTT, and OGTT, indicating that the diabetic rat model was constructed successfully (Fig. 1A, *P* < 0.01). Compared with the control group, cardiac functions were markedly deteriorated, as confirmed by the decreases in LVSP (Fig. 1B, *P* < 0.05 or *P* < 0.01)， + dp/dt_max_ (Fig. 1D, *P* < 0.05 or *P* < 0.01)_,_ and -dp/dt_max_ (Fig. 1E, *P* < 0.05 or *P* < 0.01)_,_ and the increases in LVEDP (Fig. 1C) and FBG (Fig. 1F, *P* < 0.05 or *P* < 0.01). These findings indicated marked increase in blood glucose and cardiac dysfunction in diabetic rats, suggesting that diabetes exacerbated myocardial injury, which was attenuated following OMT treatment.

### Effects of OMT on histopathology in myocardial tissues

**Fig. 2:**
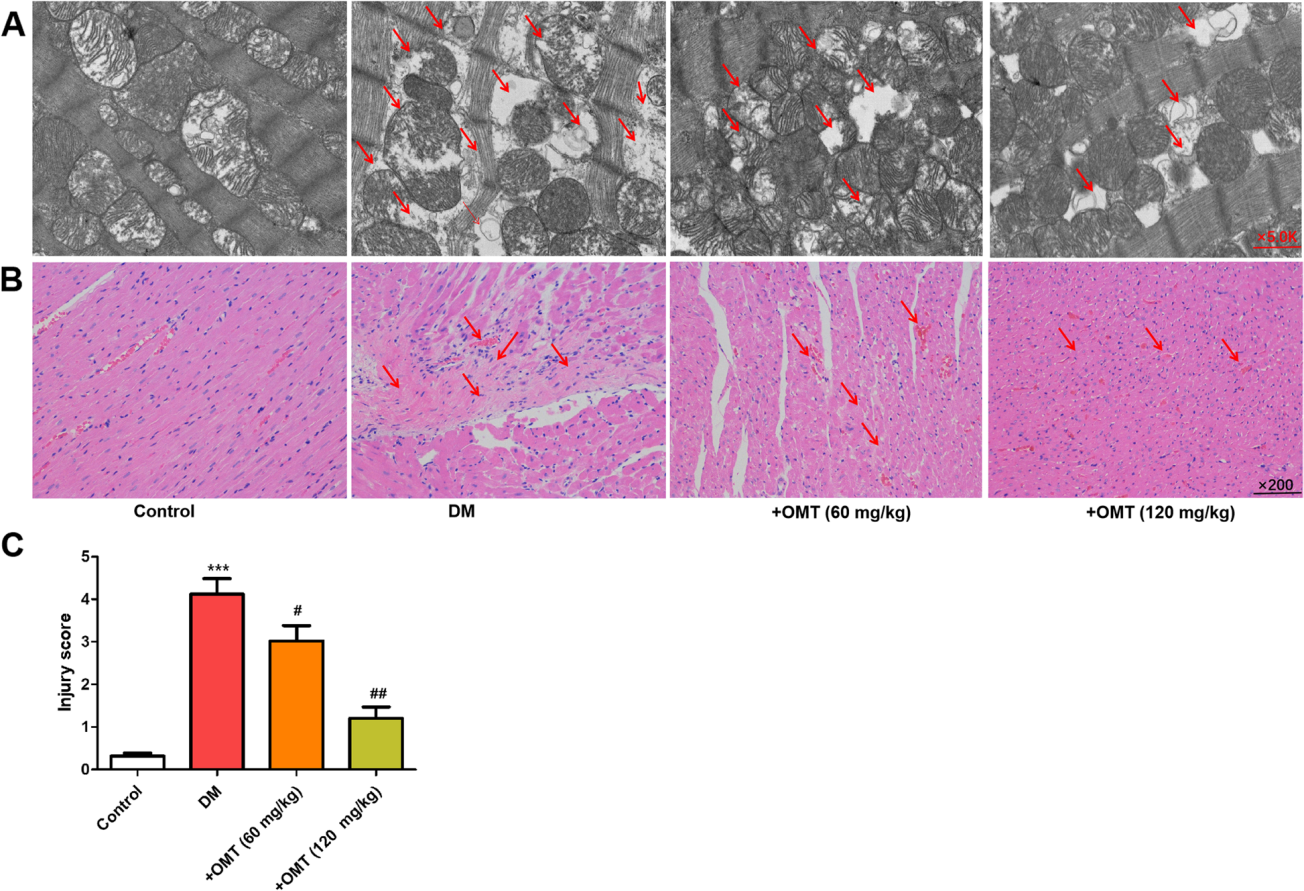
Effects of OMT on the histopathological changes and apoptosis in myocardial tissues in diabetic rats. **A** Ultrastructural changes determined using transmission electron microscopy; representative micrographs are magnified at 5000×; **B** Histopathological changes in myocardial tissues using H&E staining; representative micrographs are magnified at 200×; **C** Myocardial injury score was quantified. ^*^P < 0.05 compared with the control group. ^#^*P* < 0.05 compared with the diabetic group

Ultrastructure of myocardial tissues was assessed by electron microscopy. As demonstrated in Fig. 2A, myocardial musculature was arranged regularly and neatly, the Z-line was clear, and abundant mitochondria were observed in the cytoplasm. In the diabetic rats, ultrastructural changes demonstrated notable heterogeneous subcellular and extracellular space abnormalities in the myocardial tissues, which were attenuated by OMT.

As demonstrated in Fig. 2B, compared with the control group, diabetes caused focal confluent necrosis of the muscle fibers, with inflammatory cell infiltration, edema, and myophagocytosis, along with extravasation of red blood cells. OMT treatment significantly attenuated the aforementioned effects in myocardial tissues (Fig. 2C,, *P* < 0.05 or *P* < 0.01 or *P* < 0.001). Moreover, mild edema with a significant reduction in myocardial necrosis was observed.

### Effects of OMT on lipid peroxidation and inflammation in diabetic rats

**Fig. 3:**
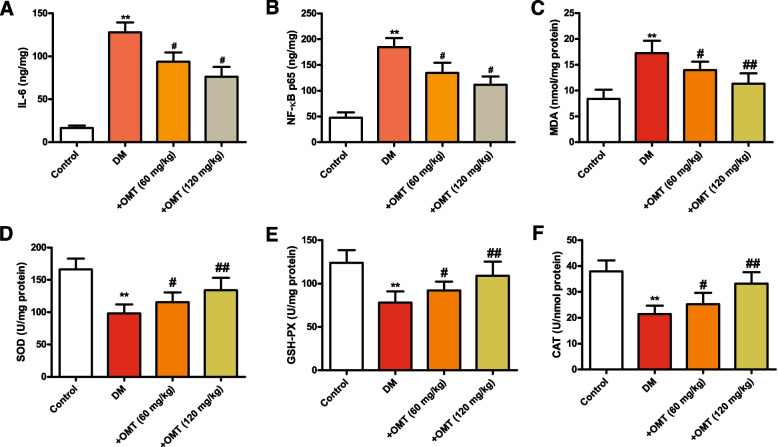
Effects of OMT on lipid peroxidation and inflammatory mediators in diabetic rats. **A** IL-6; **B** NF-κB; **C** MDA; **D** SOD, **E** GSH-Px, and (**F**) CAT. Data are expressed as the mean ± standard deviation (*n* = 6). ^*^P < 0.05 compared with the control group. ^#^*P* < 0.05 compared with the diabetic group

Colorimetric kits were used to detect the expression of inflammatory cytokines IL-6 and NF-κB in myocardial tissue. The results showed that the expression levels of IL-6 and NF-κB in diabetic rats were obviously increased compared with those in the control group (Fig. 3A and B, *P* < 0.01). Compared with diabetic rats, the expression levels of IL-6 and NF-κB in the OMT (60 mg/kg) (*P* < 0.05) and OMT (120 mg/kg) (*P* < 0.01) groups decreased in a dose-dependent manner.

Next, the expression of oxidative stress–related indicators MDA, ROS, GSH, and SOD was detected by the corresponding kits. We found that the level of MDA in the myocardium of the diabetic rats was significantly increased, while the activity levels of GSH-Px, SOD, and CAT were significantly decreased (Fig. 3C-F, *P* < 0.05 or *P* < 0.01). Compared with the diabetic rats, OMT treatment significantly reduced the levels of MDA in the myocardium and significantly increased the activity levels of GSH-Px, SOD, and CAT. These experimental results suggest that OMT reduces inflammation and lipid peroxidation.

### Effects of OMT on oxidative stress in myocardial tissues

**Fig. 4:**
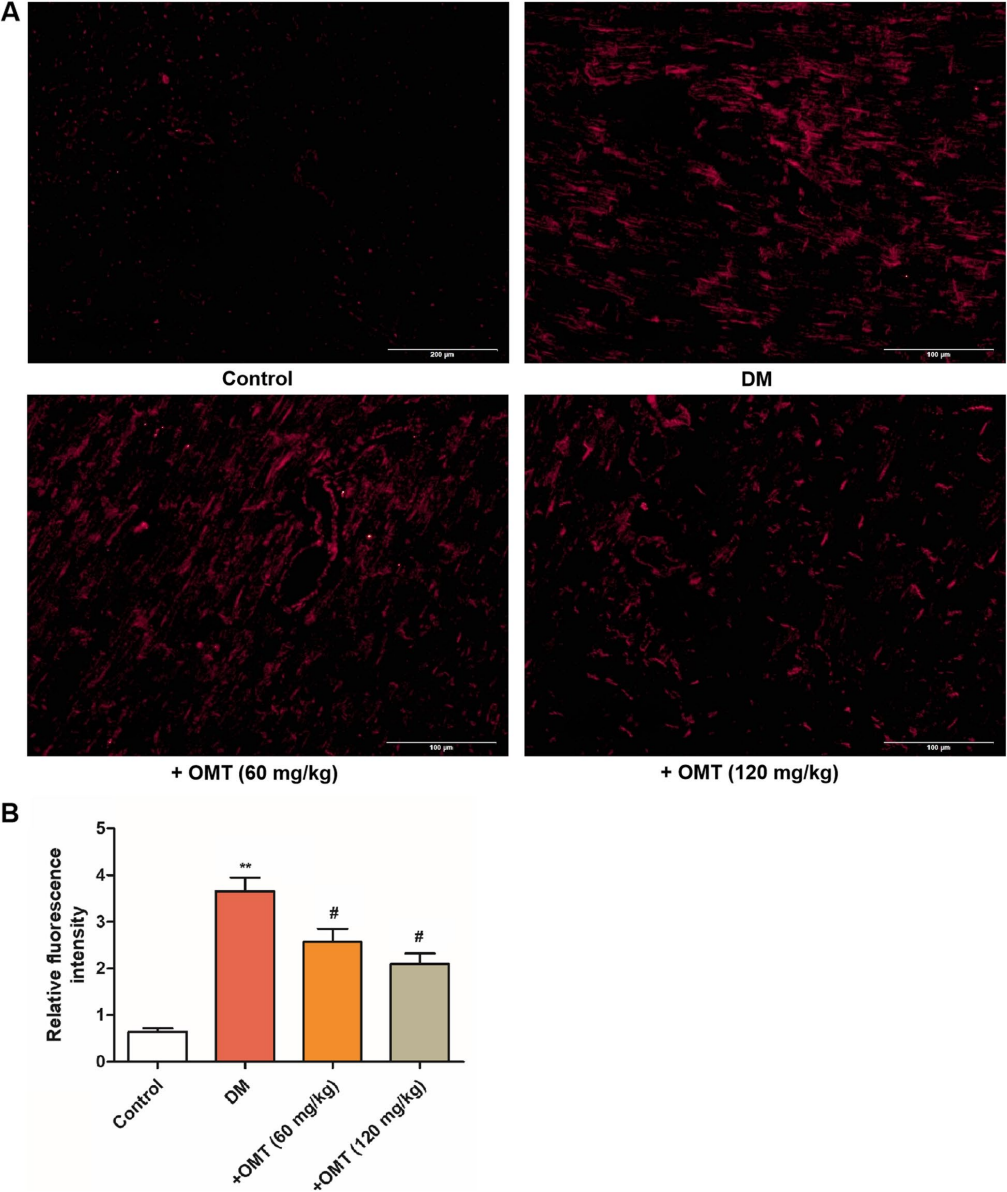
Effects of OMT on the levels of oxidative stress in the myocardial tissues of diabetic rats. **A** relative fluorescence intensity of DCFH-DA; and (**B**) the myocardial tissue slides stained using DCFH-DA. Data are expressed as the mean ± standard deviation (n = 8). ^*^P < 0.05 compared with the control group. ^#^*P* < 0.05 compared with the diabetic group

Oxidative stress is one of the critical causes of myocardial injury in diabetes [26]. Increased ROS and oxidative stress have been regarded as one of the important mechanisms contributing to diabetic cardiomyopathy. As shown in Fig. 4A and B, we found markedly increased ROS production in diabetic rats, suggesting that diabetes exacerbated myocardial oxidative stress, which was attenuated following OMT treatment (*P* < 0.05 or *P* < 0.01).

### Effects of OMT on apoptosis in myocardial tissues

**Fig. 5:**
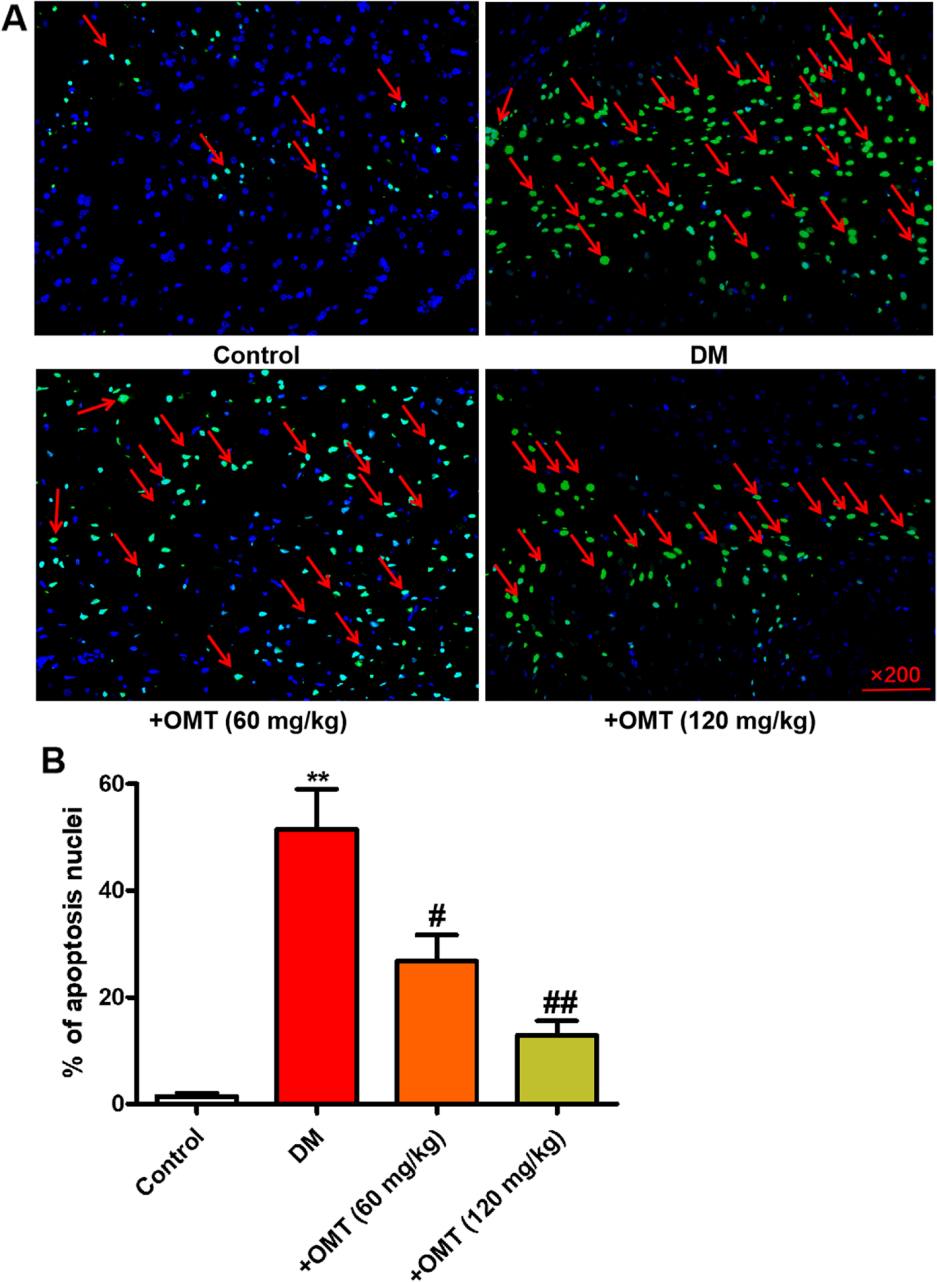
Effects of OMT on apoptosis in myocardial tissues of diabetic rats. **A** Apoptosis was detected by TUNEL staining. **B** The statistical analysis of the apoptosis percentage. ^*^P < 0.05 compared with the control group. ^#^*P* < 0.05 compared with the diabetic group

To investigate the effects of OMT on the levels of apoptosis in myocardial tissues, TUNEL assay was performed. As demonstrated in Fig. 5A and B, compared with the control group, a significant increase in the number of apoptotic cells was observed in diabetic rats (P < 0.05 or *P* < 0.01). However, OMT treatment significantly reduced the number of apoptotic cells in myocardial tissues. These results indicate that OMT may ameliorate myocardial injury partly through the inhibition of apoptosis.

### Effects of OMT on the expression levels of Nrf2 and HO-1 in myocardial tissues

**Fig. 6:**
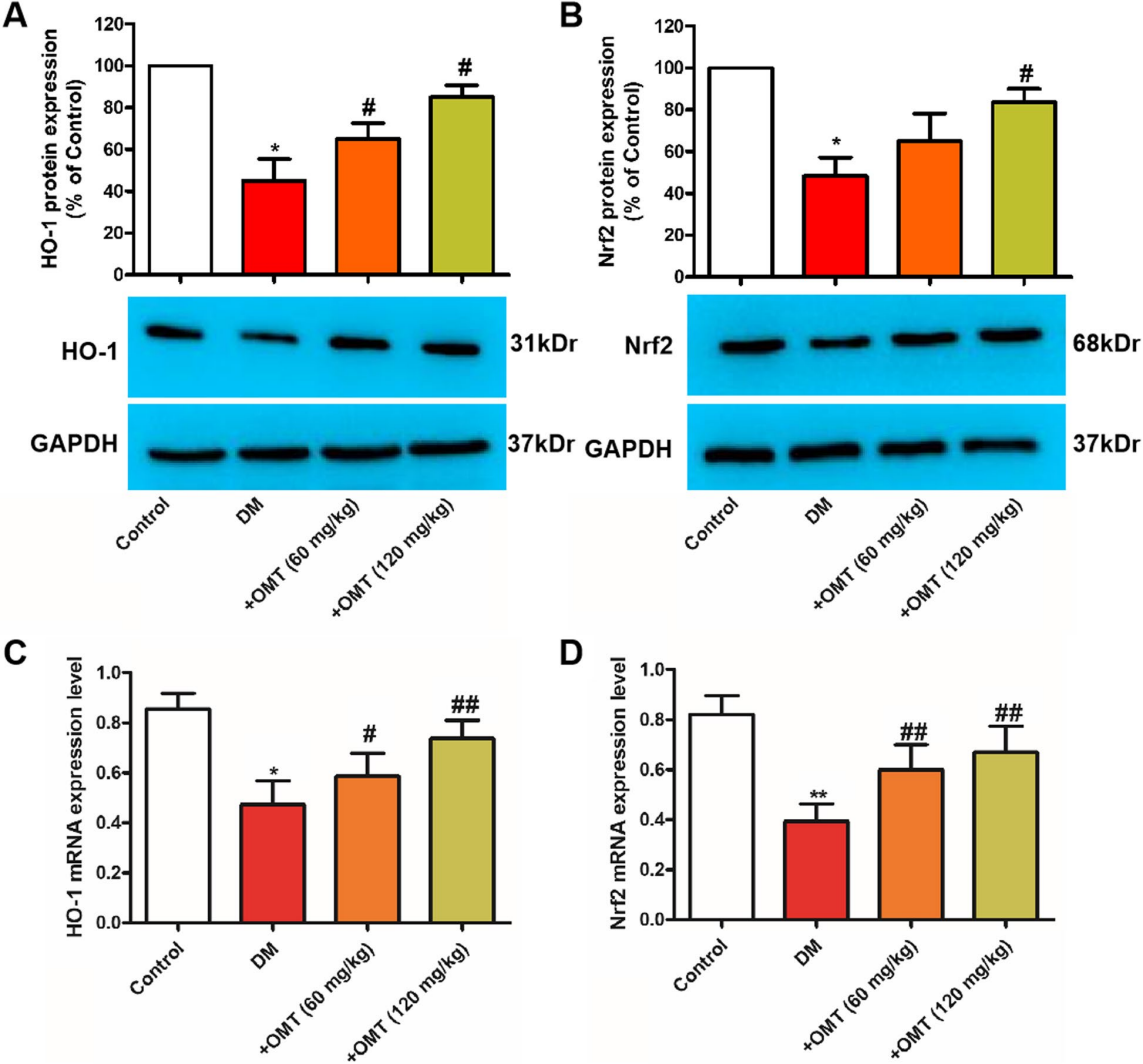
Effects of OMT on the mRNA and protein expression levels of Nrf2 and HO-1 in the myocardial tissues of diabetic rats (*n* = 3). **A** and **B** Representative images and quantitative analysis of western blotting. **C** and **D** mRNA expression levels of Nrf2 and HO-1 quantified using reverse transcription–quantitative PCR. ^**^P < 0.01 compared with the control group; ^#^*P* < 0.05 and ^##^*P* < 0.01 compared with the diabetic group

To determine whether OMT mediates the protection against oxidative stress, which is associated with the Nrf2/HO-1 signaling pathway, the levels of Nrf2 and HO-1 were determined by RT-PCR and western blot. As demonstrated in Fig. 6, the Nrf2 and HO-1 mRNA levels in the diabetic rats were significantly downregulated (*P* < 0.05). OMT treatment significantly increased the mRNA expression of Nrf2 and HO-1. Moreover, western blot analysis demonstrated that Nrf2 and HO-1 protein levels were notably upregulated, and OMT treatment decreased the levels of these oxidant-related proteins. Additionally, OMT promoted Nrf2 translocating from cytoplasm to nuclei, indicating Nrf2 functions as a major regulator in oxidative stress (Supplementary Figure, *P* < 0.05). These results demonstrate that OMT ameliorates diabetes by exerting antioxidant effects.

### Effects of OMT on the phosphorylation of JAK2 and STAT3

**Fig. 7:**
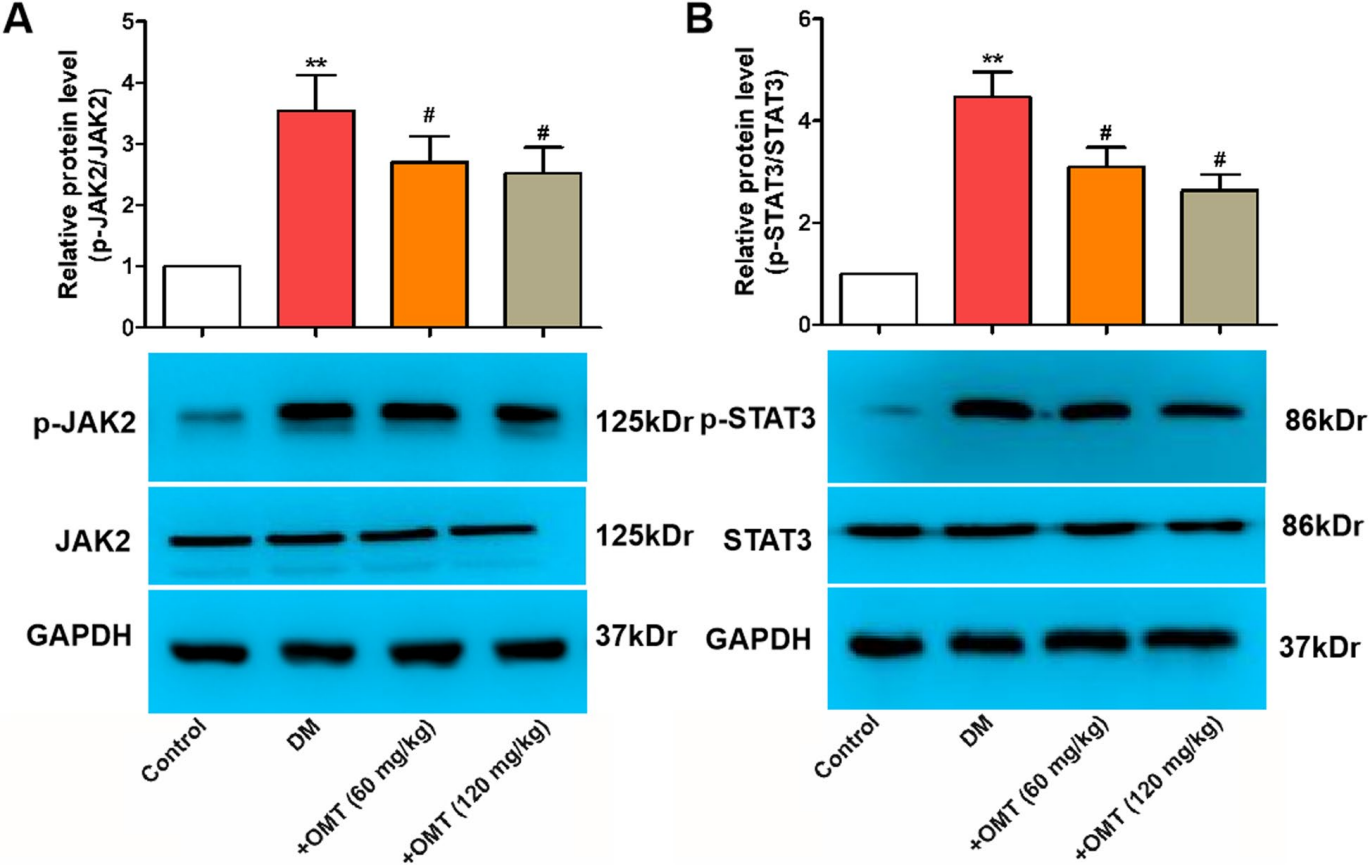
Effects of OMT on JAK2 and STAT3 protein levels in the hearts of diabetic rats. **A** and **B** Representative images of western blotting. The blots were cut prior to hybridisation with antibodies during blotting. Data are expressed as mean ± SD (n = 3). ^*^P < 0.05 compared with the control group; ^#^*P* < 0.05 and ^##^*P* < 0.01 compared with the diabetic group

The JAK–STATs signaling pathway has been shown to play a role in a variety of biological processes, including cell activation, proliferation, differentiation, autophagy, and apoptosis [27], and the activation of JAK2–STAT3 is involved in the protection of myocardial tissue [28]. However, whether the protective effect of JAK2–STAT3 is associated with amelioration of oxidative stress and the relationship of OMT with JAK2–STAT3 signaling are unclear. It has previously been reported that STAT3 is a critical downstream element of JAK2 [29]. Therefore, the effect of OMT on the JAK2/STAT3 signaling pathway was assessed. As shown in Fig. 7, there was a significant increase in the levels of phosphorylated JAK2 and STAT3 in diabetic rats (*P* < 0.05 or *P* < 0.01). However, OMT treatment significantly downregulated the levels of JAK2 and STAT3 phosphorylation. These results suggest that OMT exerts ameliorative effects on cardiac injury via suppressing JAK2 and STAT3 phosphorylation.

## Discussion

Diabetic cardiomyopathy is characterized by persistent hyperglycemia that is caused by myocardial metabolism disorders and microvascular diseases, and it ultimately leads to myocardial structural damage and cardiac dysfunction [29]. The present study demonstrated that the heart function of diabetic rats was significantly decreased and the myocardial ultrastructural damage was significantly aggravated, which further confirmed that the myocardial injury of the diabetic rat model was successfully established.

OMT is a main active gradient extracted from *Sophora flavescens* Ait. A number of studies have demonstrated that OMT exerts numerous pharmacological effects, including anti-viral, anti-inflammatory, antioxidant, antifibrotic [13–17], and antiapoptotic properties [5, 13], which has attracted a high level of attention from investigators [6, 13–16]. In the present study, diabetic rats with myocardial injury were received OMT intragastrically for 8 weeks. The results demonstrated that OMT treatment significantly decreased FBG and improved the indicators of heart function and myocardial ultrastructure. In addition, OMT treatment decreased the level of blood glucose. These results suggest that OMT exhibits protective effects on cardiac function and ultrastructural damage in a dose-dependent manner in diabetic rats.

Previous studies have demonstrated that oxidative stress and changes in the antioxidant defense systems deteriorate myocardial tissues in diabetic rats [26, 30]. Further studies have also shown that a decreased antioxidant status is an important contributor to myocardial impairment in diabetic rats [31]. Therefore, deterioration of antioxidant systems may be an important mechanism underlying increased oxidative stress in diabetes. Moreover, the content of MDA and the activities of GSH-Px, SOD, and CAT, important antioxidant enzymes in the body, reflected the degree of oxidative injury. The results of the present study demonstrated that hyperglycemia notably increased the level of MDA in myocardial tissue and markedly decreased the activities of GSH-Px, SOD, and CAT, suggesting that hyperglycemia may cause severe oxidative stress injury in diabetic rats. OMT intervention reversed the aforementioned dysfunctions, suggesting that OMT may increase the activity of antioxidant enzymes, decrease the production of lipid peroxides, and reduce oxidative stress injury in diabetic rats.

HO-1, the downstream target gene of Nrf2, is an important antioxidant protein [32]. HO-1, also known as heat shock protein 32, is a stress protein induced by multiple factors. In the presence of oxygen and NADPH, HO-1 degrades heme to produce biliverdin, free iron, and CO. Bilirubin, a derivative of biliverdin in the body, is a strong endogenous antioxidant that inhibits lipid peroxidation [33]. Therefore, HO-1 and its products exert antioxidant actions in diabetic dysfunctions [34]. Results of previous studies have confirmed that downregulation of the Nrf2/HO-1 antioxidant signaling pathway deteriorated oxidative damage in cerebral ischemia. Moreover, a previous study revealed that OMT activated the Nrf2/HO-1 pathway and attenuated the oxidative stress injury caused by renal I/R [24]. These studies highlighted the involvement of the Nrf2/HO-1 pathway in diabetes-associated myocardial disturbances. The present study further demonstrated that oxidative stress accelerated the pathogenesis of myocardial dysfunction in diabetes. The expression levels of Nrf2 and HO-1 in the myocardial tissue were notably increased, and these expression levels decreased following treatment with OMT in a dose-dependent manner. This finding suggests that OMT may enhance the antioxidant capacities via upregulation of the Nrf2/HO-1 signaling pathway in diabetic rats.

Inflammation has been reported as one of the early events that accelerates the progression of diabetic myocardial injury. Our findings showed that OMT significantly lowered proinflammatory cytokines, showing significant anti-inflammatory activity. This result is consistent with the pronounced antioxidant effect of OMT, since oxidative stress is the main trigger for the release of proinflammatory cytokines [35, 36]. In fact, oxidative stress and inflammation are inextricably linked, generating and amplifying each other [35]. Numerous studies have demonstrated that Nrf2 downregulates NF-κB, a transcription factor that affects multiple cytokines, including IL-6 [37]. Therefore, the decreased IL-6 level in the OMT-treated group may be related to the enhanced expression of Nrf2 protein and the subsequent decrease of NF-κB level.

Increasing amount of evidence indicate that multiples of mechanisms involved in cell apoptosis in myocardial injury, including activation of SIRT1-Nrf2 [38], LKB-1/AMPK/Akt pathway and suppression of GSK-3β and p38α/MAPK pathway [39] and so on. And our understanding of diabetes-associated myocardial injury events at apoptosis has advanced substantially. The JAK/STAT pathway is involved in many important biological processes such as cell proliferation, differentiation, apoptosis and immune regulation [40] and several reports have proposed that JAK/STAT signalling is associated with cardiac dysfunction in diabetes [41, 42]. In diabetes, IL-6 inhibits the viability and apoptosis of pancreatic beta-cells via modulation of microRNA-22 in the JAK/STAT signaling pathway [43]. Previous studies have demonstrated that high glucose induces the activity of the JAK/STAT signaling pathway, leading to TGF-β1 activation and the subsequent increase in cardiac fibroblasts [44]. Our results suggest that diabetes caused notable increases in the phosphorylation of JAK2 and STAT3 and the levels of TGF-β1. Previous studies have demonstrated that IL-6 activates the JAK/STAT signaling pathway; thus, the pronounced increases in p-JAK2 and p-STAT3 may be attributed to the elevated level of proinflammatory cytokines in diabetic rats [45]. Conversely, the overt anti-inflammatory activity of OMT revealed an inhibitory effect on p-JAK2, p-STAT3, and TGF-β1 [46]. OMT was found to downregulate JAK2 and STAT3 phosphorylation in multiple pathophysiological states [47, 48]. Thus, the JAK2/STAT3 pathway may be considered to be of vital importance in ameliorating the effects of OMT against diabetic cardiomyopathy.

However, there were some limitations regarding the application of OMT for myocardial injury in diabetic rats. For example, in vitro investigations into the effects of OMT are required. In addition, to determine whether the JAK2/STAT3 pathway mediates the cardioprotective activity of OMT, the use of an inhibitor or short hairpin RNA is required, and further studies must be carried out.

Collectively, this study provides evidence that the cardioprotective effects of OMT may be associated with the inhibition of the Nrf2/HO-1 and JAK/STAT pathways and an increase in the activity of antioxidant enzymes in diabetic rats.

## Supplementary Information

**Additional file 1.**

## Data Availability

The datasets used and/or analyzed during the current study are available from the corresponding author on reasonable request.
