# Supplementary material for: Oxymatrine ameliorates myocardial injury by inhibiting oxidative stress and apoptosis via the Nrf2/HO-1 and JAK/STAT pathways in type 2 diabetic rats
Source: BMC Complement Med Ther. 2023 Jan 3;23:2. doi: 10.1186/s12906-022-03818-4 (PMC9808977; doi:10.1186/s12906-022-03818-4)

Nrf2 regulates the expression of multiple cellular defense proteins through the antioxidant response element (ARE) and also functions as a major regulator of cellular lipid disposition in the heart (1,2). Here, we found that the nuclear Nrf2 level was decreased in diabetic rats but was clearly increased by OMT treatment. In contrast, the cytosolic increased Nrf2 was restored to normal levels after OMT treatment thus suggesting that OMT promoted Nrf2 translocating from cytoplasm to nuclei (Supplementary Figure)


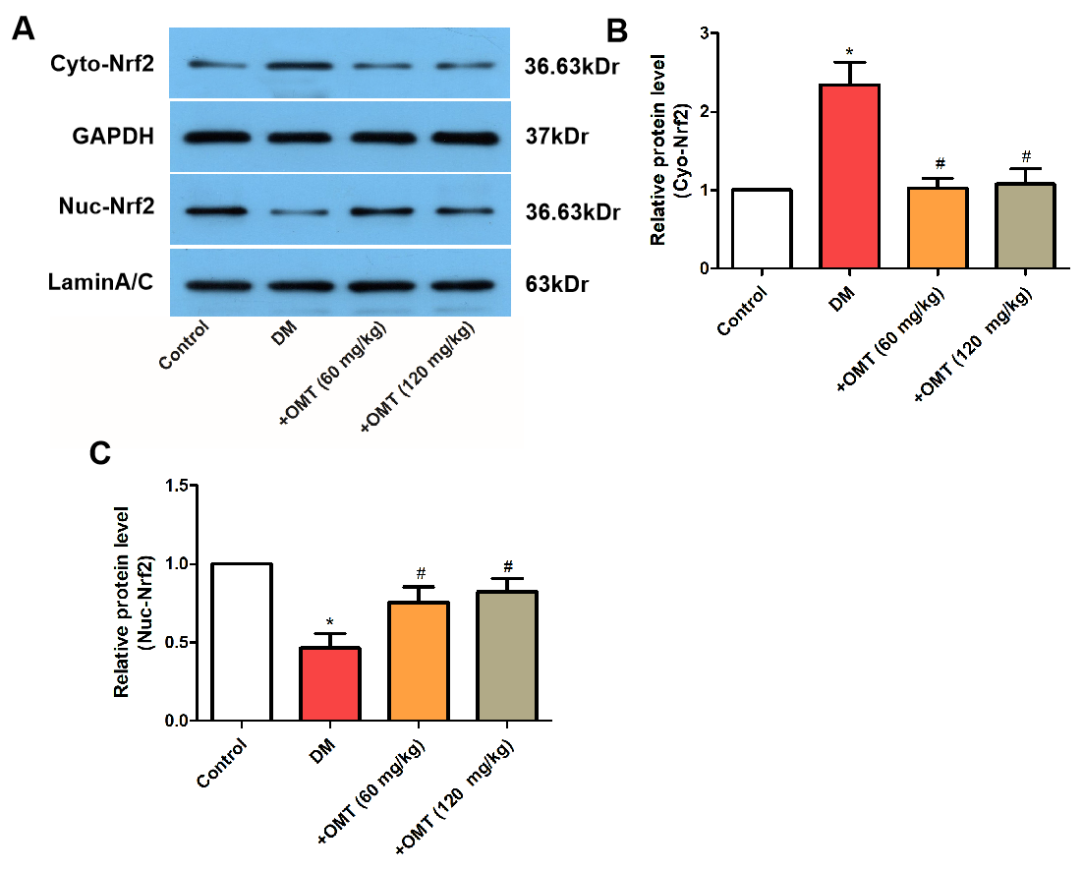


Supplementary Figure. Cytoplasmic and Nuclear proteins were recovered from lysed myocardial tissue of diabetic rats treated with OMT (60 mg/kg), or OMT (120 mg/kg) (Supplementary Figure A), and were subjected to western blotting and quantified by using Image J software (Supplementary Figure B and C). Data are means ± SD. ^*^*P* < 0.05 compared with the control group. ^#^*P* < 0.05 compared with the diabetic group.

**Reference**

1. Luo J, Yan D, Li S, Liu S, Zeng F, Cheung CW, Liu H, Irwin MG, Huang H, Xia Z. [Allopurinol reduces oxidative stress and activates Nrf2/p62 to attenuate diabetic cardiomyopathy in rats.](https://pubmed.ncbi.nlm.nih.gov/31856386/) J Cell Mol Med. 2020 Jan;24(2):1760-1773.
2. Zhang B, Zhai M, Li B, Liu Z, Li K, Jiang L, Zhang M, Yi W, Yang J, Yi D, Liang H, Jin Z, Duan W, Yu S. [Honokiol Ameliorates Myocardial Ischemia/Reperfusion Injury in Type 1 Diabetic Rats by Reducing Oxidative Stress and Apoptosis through Activating the SIRT1-Nrf2 Signaling Pathway.](https://pubmed.ncbi.nlm.nih.gov/29675132/) Oxid Med Cell Longev. 2018 Feb 20;2018:3159801.

Figure 5


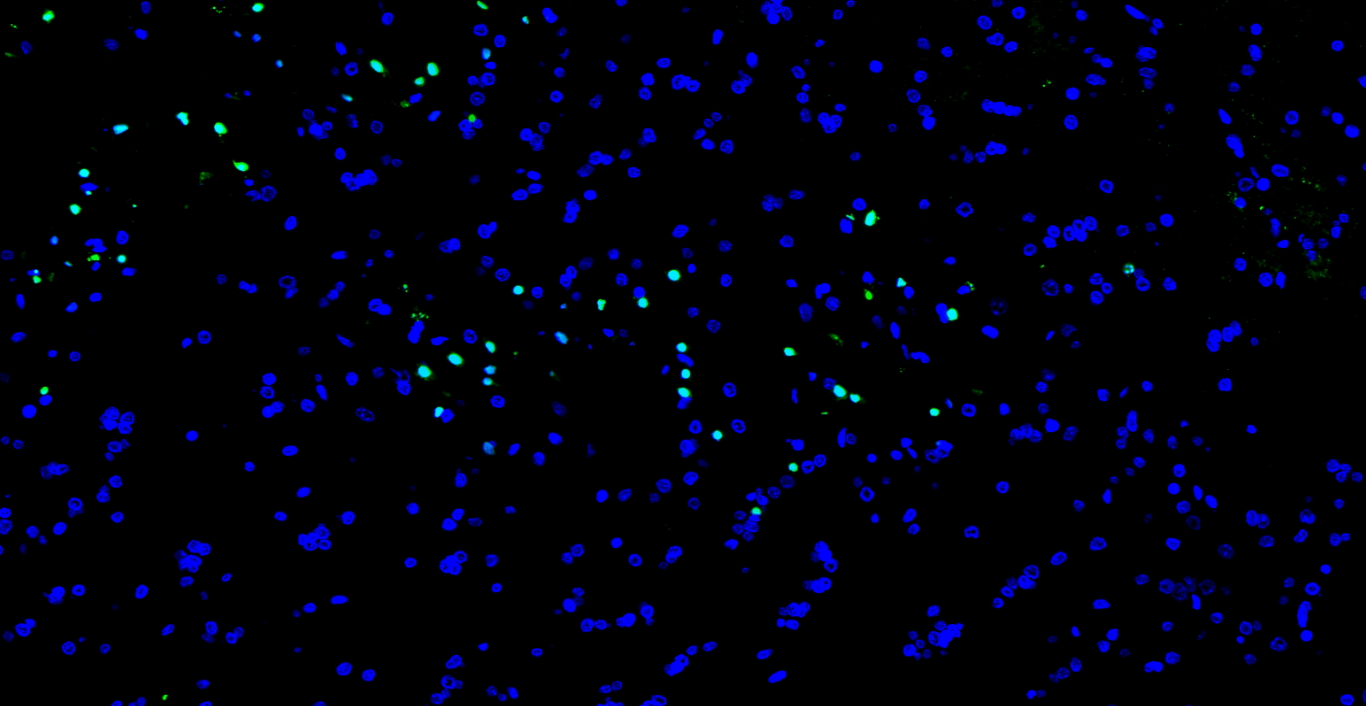


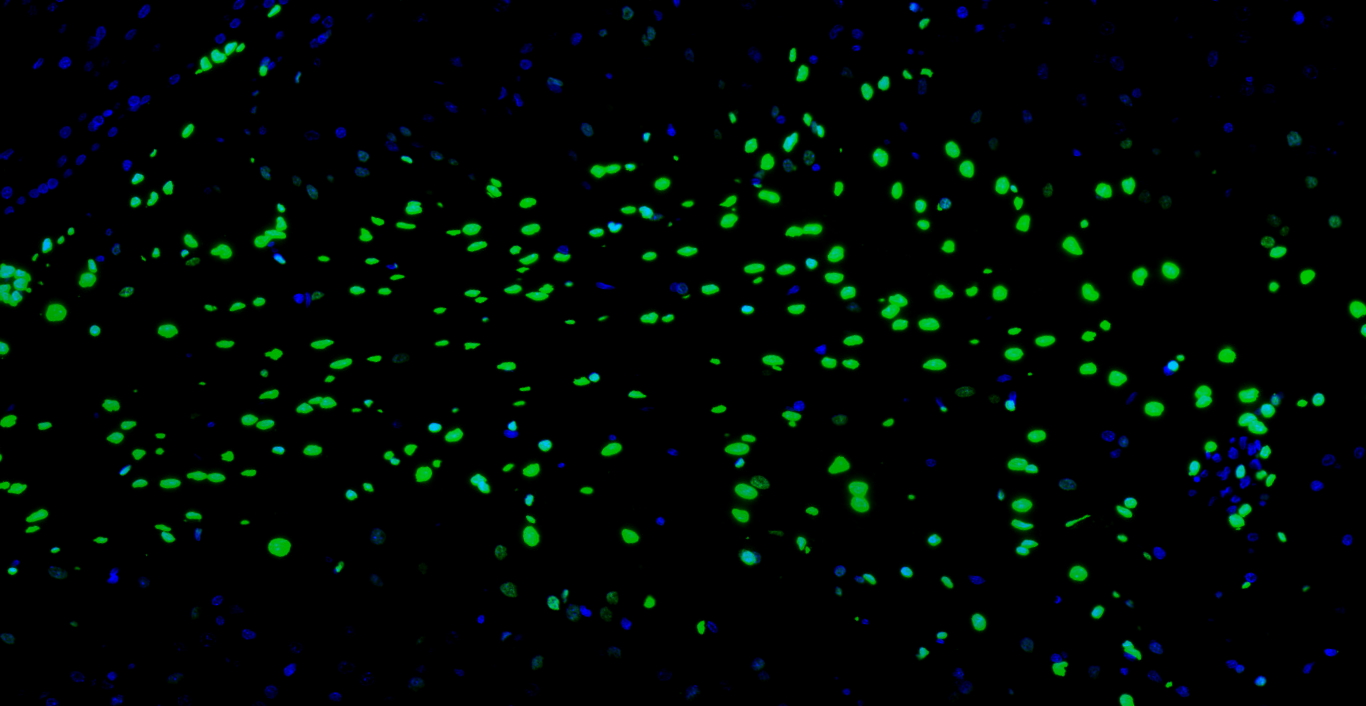


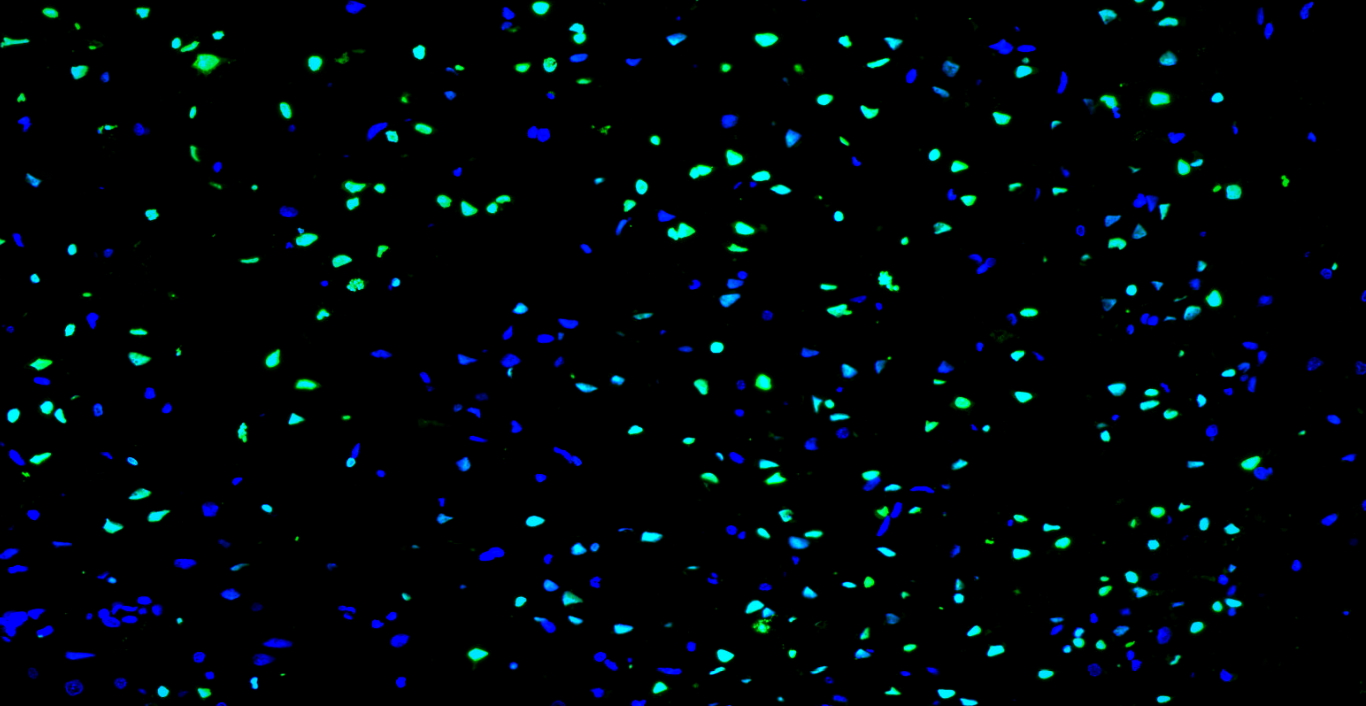


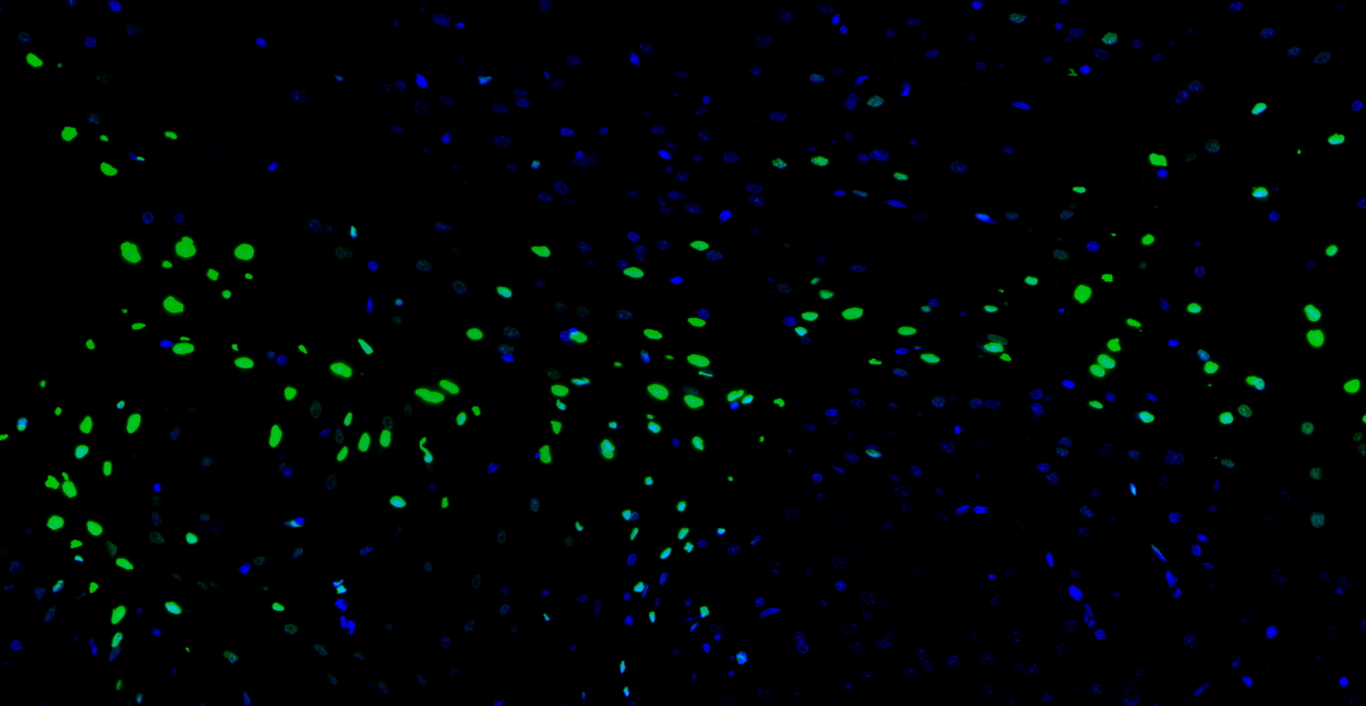


Figure 6


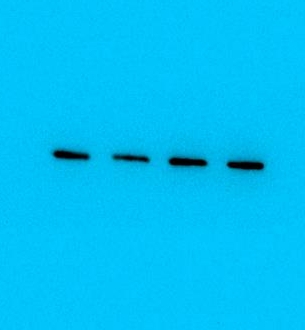


HO-1


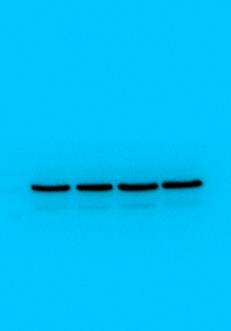


GAPDH


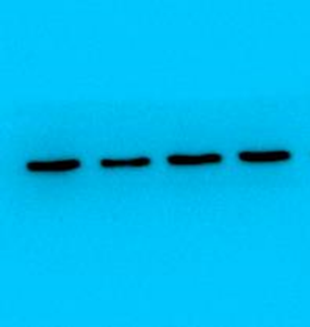


Nrf2


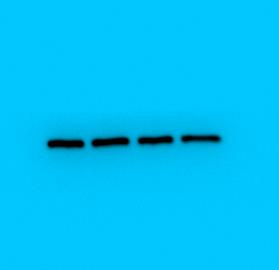


GAPDH

Figure 7A


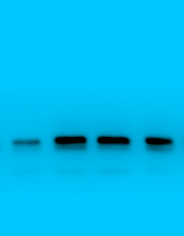


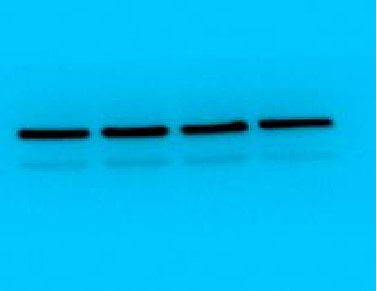


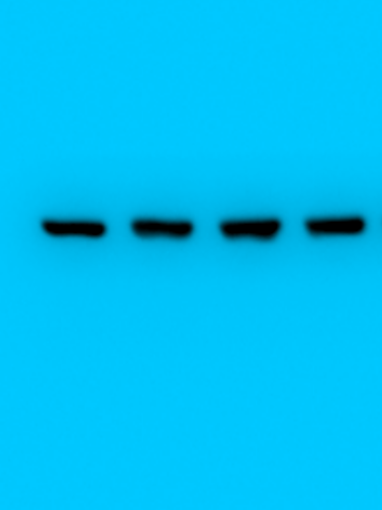


Figure 7B


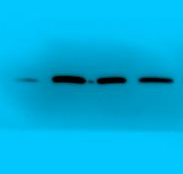


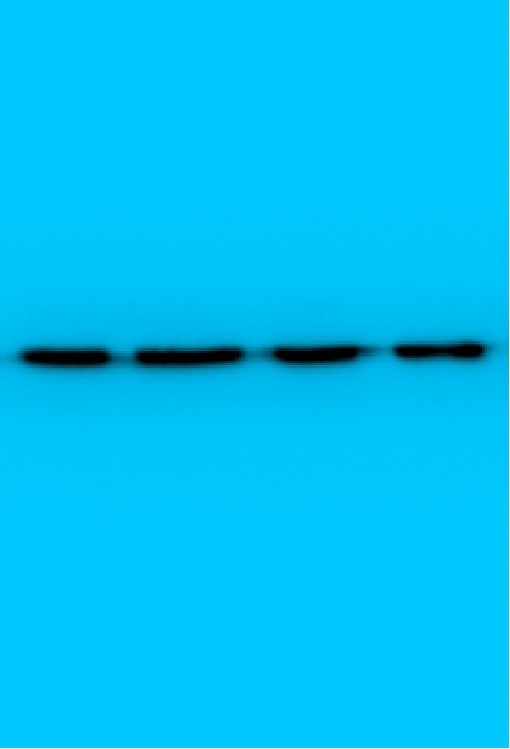


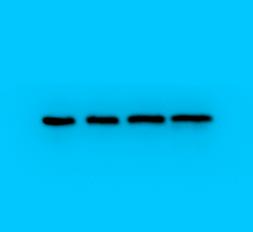


Supplementory figure


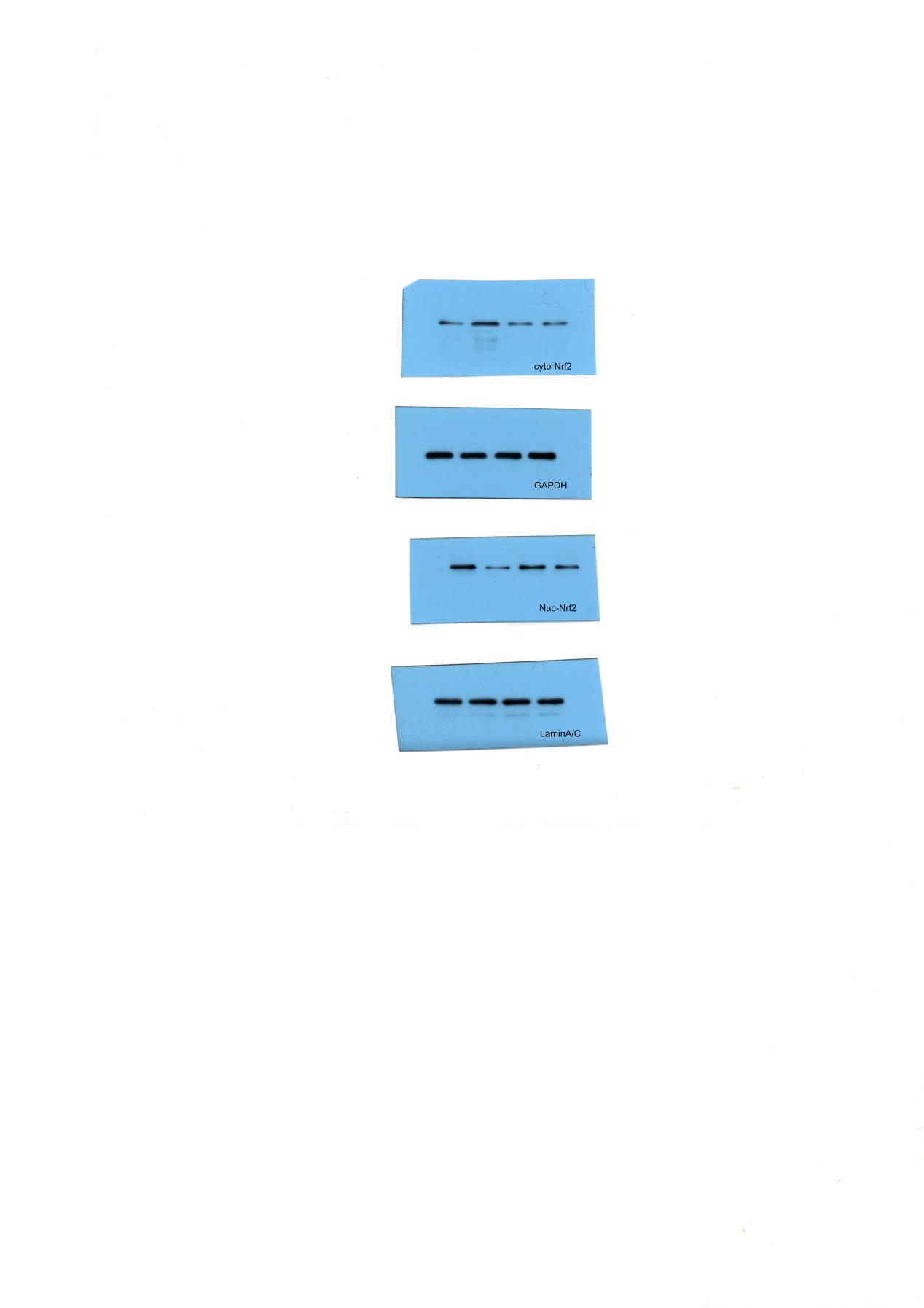

Supplement: Supplementary file 1 — Additional file 1. [file 12906_2022_3818_MOESM1_ESM.zip › 2-final edition-supplemetory results.docx]
