# Supplementary figures and images for: Oxymatrine ameliorates myocardial injury by inhibiting oxidative stress and apoptosis via the Nrf2/HO-1 and JAK/STAT pathways in type 2 diabetic rats
Source: BMC Complement Med Ther. 2023 Jan 3;23:2. doi: 10.1186/s12906-022-03818-4 (PMC9808977; doi:10.1186/s12906-022-03818-4)

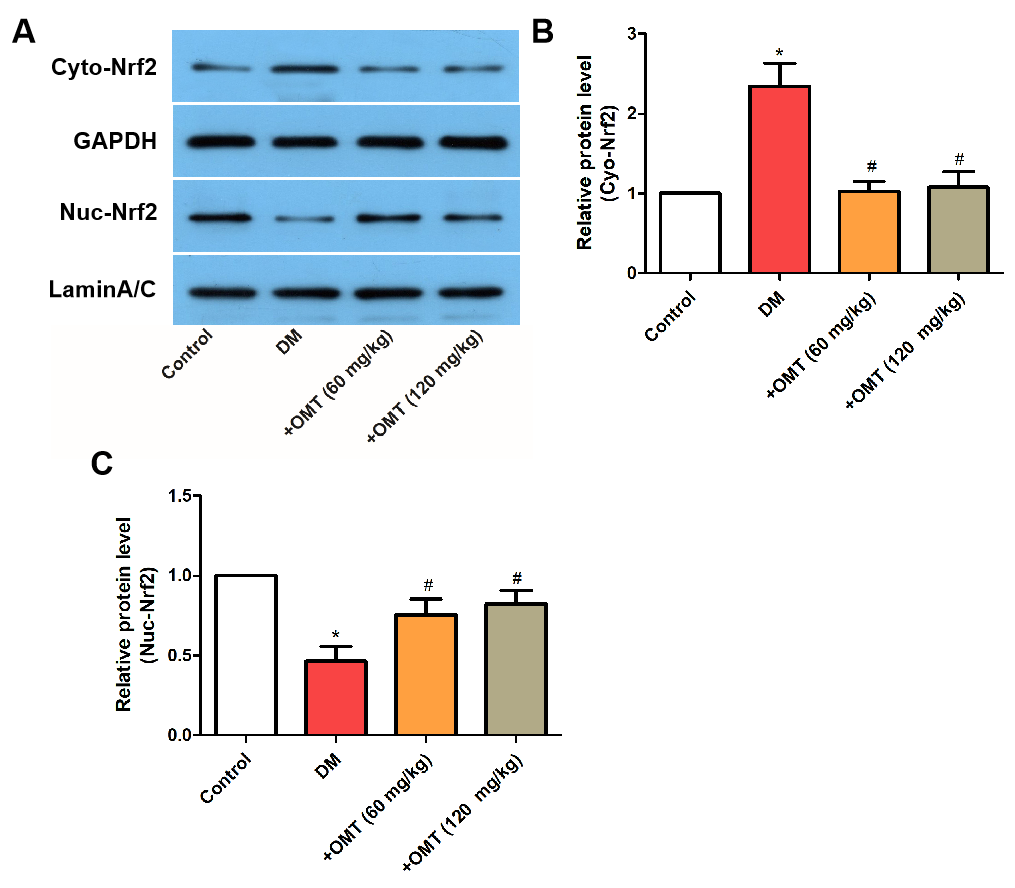

Supplement: Supplementary file 1 — Additional file 1. [file 12906_2022_3818_MOESM1_ESM.zip › supplementory figure.tif]
